# Treatment of 95 post-Covid patients with SSRIs

**DOI:** 10.1038/s41598-023-45072-9

**Published:** 2023-11-02

**Authors:** Carla P. Rus, Bert E. K. de Vries, Ingmar E. J. de Vries, Idelette Nutma, J. J. Sandra Kooij

**Affiliations:** 1 Independent Researcher, The Hague, The Netherlands; 2 Donders Institute, Radboud University, 6525 EN Nijmegen, The Netherlands; 3 Centre for Mind/Brain Sciences (CIMeC), University of Trento, 38068 Rovereto, Italy; 4 Department of Psychiatry, Amsterdam UMC/VUMC, 1081 HJ Amsterdam, The Netherlands; 5 PsyQ, 2593 HR The Hague, The Netherlands

**Keywords:** Diseases, Medical research, Neurology, Diseases of the nervous system, Molecular neuroscience, Neural circuits, Synaptic transmission, Transporters in the nervous system

## Abstract

After Covid-19 infection, 12.5% develops post-Covid-syndrome (PCS). Symptoms indicate numerous affected organ systems. After a year, chronic fatigue, dysautonomia and neurological and neuropsychiatric complaints predominate. In this study, 95 PCS patients were treated with selective serotonin reuptake inhibitors (SSRIs). This study used an exploratory questionnaire and found that two-thirds of patients had a reasonably good to strong response on SSRIs, over a quarter of patients had moderate response, while 10% reported no response. Overall, patients experienced substantial improved well-being. Brainfog and sensory overload decreased most, followed by chronic fatigue and dysautonomia. Outcomes were measured with three different measures that correlated strongly with each other. The response to SSRIs in PCS conditions was explained by seven possible neurobiological mechanisms based on recent literature on PCS integrated with already existing knowledge. Important for understanding these mechanisms is the underlying biochemical interaction between various neurotransmitter systems and parts of the immune system, and their dysregulation in PCS. The main link appears to be with the metabolic kynurenine pathway (KP) which interacts extensively with the immune system. The KP uses the same precursor as serotonin: tryptophan. The KP is overactive in PCS which maintains inflammation and which causes a lack of tryptophan. Finally, potential avenues for future research to advance this line of clinical research are discussed.

## Introduction

Post-Covid-syndrome (PCS) is a multisystem disease with more than 200 different symptoms^1^. As this condition has only been recognized for three years, much is still unknown and new symptoms are still emerging. There are over 100 million PCS patients worldwide. This figure is based on an extrapolation of figures from the United Kingdom^2^. As a result of the associated severe disability PCS is a substantial health problem with major personal, societal and economic consequences. The impact of PCS on patients is disruptive: for their physical and mental health; household and family, bringing up of children, postponement of having children, social and leisure activities; education, work and income. This also has serious economic impacts. Bach estimated an annual cost of 230 billion dollars in the USA, assuming 4 million people out of work due to PCS^3^.

The main PCS symptoms are inter alia: ‘brainfog’ (consisting of ‘cloudy thinking', concentration and memory problems), headache, sensory overload (overstimulation), severe fatigue, dyspnoea, post-exertional malaise (PEM), dysautonomia including postural orthostatic tachycardia syndrome (POTS), palpitations, disturbed sleep, muscle pain, mast cell activation syndrome (MCAS), intestinal problems, decrease in smell and tinnitus. These are mainly neurological and neuropsychiatric in origin. In many PCS patients, no clear pathophysiological substrate is found on usual (blood) examination. However, autonomic dysfunction is demonstrated with the tilt table test^4^. Here, the supine patient is raised to an angle of about 70%, while heart rate and blood pressure are monitored. Thus, POTS and other signs of dysautonomia can be diagnosed. Small fibre neuropathy is also sometimes found^5^.

The literature reports as cause of PCS: neuroinflammation, increased pro-inflammatory cytokines^6,7^, autoimmune reactions, hypoxia due to microclots, fibrin-amyloid microclots and reactivation of herpes viruses such as Epstein-Barr virus^8^. Hypometabolic areas are also found in the pons (a part of the brain stem)^9^. The vagus nerve also arises from the pons. This cranial nerve may dysfunction in PCS, leading to dysautonomia^1^. The pons is the location of the origin of the serotoninergic system. From there, axons are sent throughout the CNS^10^.

Early on, we recognized similarities between the symptoms of PCS and chronic fatigue syndrome (ME/CFS)^11^. ME/CFS is known to increase pro-inflammatory cytokines in the brain^12^ and reduce hypothalamic–pituitary–adrenal (HPA) axis function over time^13^. The HPA axis provides the release of glucocorticoids (GCs). GCs act on almost all types of immune cells and perform evident immunosuppressive and anti-inflammatory functions^14–18^. According to the literature, it appears that in PCS patients, on average, cortisol levels are measured to be 50% of normal^14,19^. This supports our hypothesis that a disturbed HPA axis is also present in PCS.

Selective serotonin reuptake inhibitors (SSRIs) have at least three ways to influence the immune system. In the first place by modulating the afore-mentioned HPA axis^14–18^. They do this by activating serotonin- and norepinephrine-neurotransmitter systems. Neurotransmitters are substances that transmit signals between nerve cells and brain nerve cells (neurones)^20^. Although an SSRI is normally indicated for depression and anxiety disorders^16,21^, serotonin is found in many parts of the body: in the digestive system^22,23^, blood platelets^24^ and throughout the whole central nervous system (CNS)^25^. So an SSRI has far reaching impact in the body. An SSRI makes serotonin and norepinephrine reuptake into the presynaptic neuron less likely, allowing these extra neurotransmitters in the synaps to transmit their signal to the postsynaptic neuron for longer^16,20,21^.

In the second place SSRIs can potentially influence the immune system through interaction with the kynurenine pathway (KP). The KP has the function to create an important energy cofactor: nicotinamide adenine dicleotide (NAD +). There is an extensive interaction between the KP and the immune system^26–28^. The KP is overactive in many inflammations^26,29^ as well in PCS^27,28,30^ and contributes to the maintenance of inflammation. Both the serotonin pathway and the KP use the same precursor tryptophan, an essential amino acid. In the event of a deficiency of this precursor, which is the case with PCS^30–32^, the serotonin pathway activated by SSRIs could be regarded as a competitor of the KP. (See Fig. 6 in section “Potential mechanisms of action of SSRIs”).

In the third place some SSRIs have additional anti-inflammatory effects, such as inhibition of sphingomyelinase acid (ASM)^33^ or are an sigma1 receptor agonist involved in reduction of virus replication and reactivation of herpes viruses such as Epstein-Barr virus^33–35^. An agonist is a stimulator of the receptor, in contrast to an antagonist which inhibits.

As regards empirical evidence:** i**n a review of 14 clinical trials, 10 studies found that an SSRI or a serotonin and norepinephrine reuptake inhibitor (SNRI) given during a Covid-19 infection reduced the severity of the infection^36^. Another study found that an SSRI given during Covid-19 infection could prevent PCS^37^. Treatment duration with an SSRI was only 10 to 14 days in all these studies. In existing PCS, only one study is known of treatment with an SSRI, with depression as the indication^38^. In that study of 60 patients, depression improved in 92%. The effect on PCS symptoms was not reported^38^. To date, there is no known effective medicinal interventions in PCS yet, other medications are under investigation^39^ Research with SSRIs is currently lacking. This is the reason the first author wrote an article in the Dutch newspaper NRC on Dec. 17, 2020 suggesting that an SSRI could possibly be used in the treatment of PCS. After publication, increasing numbers of PCS patients reported us wanting to try treatment with an SSRI. Given the surprisingly positive reports from those patients, such as: “I have my old life back”, we have continued to recommend considering an SSRI in PCS ever since.

Owing to the potential importance of treatment to the large group of PCS patients, we decided in late 2022 to investigate the effect of treatment with an SSRI on PCS.

## Method

### Ethical declaration

The Medical Ethics Review Committee of Amsterdam University Medical Centers has reviewed the research, nr. 2023.0358. Based on the protocol and the documents submitted, the committee concludes that the design of the study meets the requirements arising from applicable laws and regulations, including ECTR, MDR or IVDR, WGBO (Medical Treatment Contract Act) and the AVG (General Data Protection Regulation). All methods were performed in accordance with the relevant guidelines and regulations.

The Medical Ethics Review Committee of Amsterdam University Medical Centers is registered with the US Office for Human Research Protections (OHRP) as IRB00013752. The FWA number assigned to Amsterdam UMC is FWA00032965.

### Treatment

Patients who were interested in treatment with an SSRI and their general practitioners (GP) were informed by e-mail about the experimental nature of the treatment, the possible response to SSRIs in PCS, which SSRIs could be used, the dosage, the titration and the possible side effects. The information for patients and physicians was also posted on the website sepsis-en-daarna.nl/en/.

We did not treat the patients ourselves but advised them to consult with their own (primary) physician about treatment with an SSRI. It was always important to emphasize that the SSRI in PCS was advised for other working mechanisms than for depression or anxiety symptoms. Of the SSRIs, sigma1 receptor agonists such as fluvoxamine, citalopram, escitalopram and fluoxetine were advised preferentially. This is because these drugs can reduce elevated pro-inflammatory cytokines^34^. We also recommended venlafaxine, which is not an SSRI, but an SNRI. From a dosage of 150 mg daily, it also acts through the dopaminergic system. It was advised to start with a low dose and depending on response and side effects, to titrate upwards carefully, until an acceptable dose was reached. We used the dose for depression as a guideline. If there were many side effects, we titrated the SSRI even more slowly, for example, adding a drop of 2 mg citalopram every two weeks. The final dosages of the SSRIs varied. Some patients ended up on 30 mg citalopram, others only on 5 mg. The same variation was also true for the other SSRIs. It has remained customized work. In case of persistent serious side effects, we advised patients and their physicians to use another SSRI, possibly after having a pharmacogenetic profile created first (Table 1).

**Table 1:** Final doses of the various SSRIs in mg.

SSRI	Nr of patients (n = 95)	Mean dose	std	Lowest dose	Highest dose
Citalopram	46	19.3	8.4	5	40
Venlafaxine	17	150.0	79.5	37.5	225
Fluvoxamine	15	114.2	66.0	50	300
Escitalopram	10	13.0	6.7	5	20
Fluoxetine	2	17.5	3.5	15	20
Sertraline	4	62.5	25	50	100
Paroxetine	1	10	0	10	10

### Interactions with other medications

We did not prescribe the medicines ourselves but we gave advice, so the final responsibility remained with the prescribing physician. Many patients had been prescribed mirtazapine, nortriptyline or quetiapine to help them sleep. All three increase—like SSRIs—the QTc interval, the first two of which also carry a risk of a serotonergic syndrome. We recommended first phase out those three. The SSRI often helps people sleep better. If not, we recommend promethazine syrup (first generation phenothiazine), a sedative Histamine1 (H1) antagonist instead. To avoid a possible slightly improvement of the effect of the SSRI^40^, we kept the doses as low as possible (5 mg to 15 mg at night). Many people used desloratadine (H1 antagonist) because of allergies and theoretically that may also improve the effect of an SSRI. Omeprazole (CYP2C19 inhibitor) increases the serotonin level. But we stopped increasing the SSRI if there were too many side effects and backed off if necessary. People who used omeprazole (n = 5), received: venlafaxine 37.5 mg; paroxetine 10 mg; citalopram 2 × 20 mg; escitalopram 5 mg. Aspirin and diclofenac (increasing risk of bleeding) were also advised to greatly reduce or phase out. Use of solifenacin and salmeterol (fluticasone) (both increase QTc interval) could not be tapered and were combined with fluvoxamine 100 mg and 75 mg, respectively.

### Inclusion and exclusion

**Figure 1:**
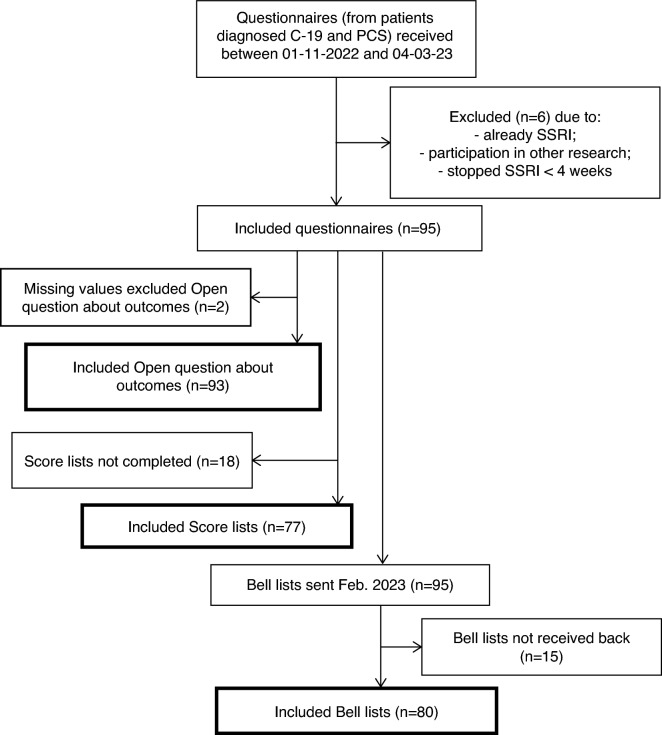
Flow chart of inclusion and exclusion.

From November 1st 2022 onwards PCS-patients who contacted us, or had contacted us before and started an SSRI earlier, were asked to return a completed questionnaire with informed consent for pseudonymised processing of all data. They also gave permission to be contacted again with additional questions for the purpose of the study. To achieve sufficient volume, we aimed at receiving 100 questionnaires. After we received 101 questionnaires on March 4th 2023 we closed the field phase of this study. We started with the initial 101 questionnaires, without any selection, so regardless of demographic characteristics, the seriousness of their illness or the outcomes of the treatment. We included only patients with a proven transient Covid-19 infection, demonstrated by PCR or antigen test, followed by symptoms of PCS, who were treated with an SSRI. Three patients had developed PCS after Covid-19 vaccination. All three had a Covid-19 infection just before. The diagnosis of PCS following Covid-19 infection was made by the GP and/or medical specialist based on the clinical pattern of symptoms. See Fig. 1.

Exclusion criteria for participation were: long-standing use of an SSRI for depression/anxiety disorder (2x), concurrent participation in other drug study (1x), and stopping within 4–6 weeks of starting the SSRI due to side effects (3x). This left a population of 95 patients, all Dutch citizens. Patients who had discontinued an SSRI after more than 4 weeks because of side effects, or had not increased the dose because of side effects, were included.

### Questionnaire

All questionnaires received were processed pseudonymously. Questionnaires were stored by number and the data were entered into Excel files. The key with names and numbers was carefully managed and used only to obtain further information if necessary for the purpose of the study.

The questionnaire contained two parts. Part A reported on the situation before, and Part B on the situation after treatment with an SSRI.

Part A included both open and closed questions about demographics, PCS symptoms, medical history, vitamins D and B_12_ status, medication use, nutritional supplements, other previous treatments and their outcomes. If vitamin D and B_12_ status was unknown, we asked them to have this tested by their physician and to complement if necessary before starting the SSRI. Vitamin D can regulate the immune system and low levels of vitamin D are associated with PCS^41,42^. Vitamin B_12_ can help balance immune responses to better fight viral infections^43^. We also asked about the time lag between Covid-19 and PCS. The questionnaire also contained a score for the severity of 8 common PCS complaints in both part A and part B (see below). Participants were requested to complete part A immediately upon receipt. The open question about PCS complaints gave the patients the opportunity to report previously undescribed PCS symptoms. Existing questionnaires such as the Chalder Fatigue Scale or the Canadian Consensus Criteria^44^, both developed for ME/CFS, were deemed less suitable for this purpose^11^.

Part B was completed at least 4 to 6 weeks after starting with an SSRI. In Part B, participants were asked which SSRI was used, in what dosage they started, the titration and the final dosage and about the onset of any response and side effects. In an open question about outcomes, we asked about any reduction in symptoms. The Scoring list for the severity of 8 complaints was repeated in part B.

### Control retrospective reporting

61 patients—who under our guidance—had already started an SSRI before November 1, 2022, were asked to complete part A of the questionnaire retrospectively. For this group, the analysis used the first e-mail to us with the description of the original complaint to identify the baseline measurement. In all cases, the PCS complaints as described in the questionnaire matched the summary of the complaints in the registration email. As a result, for this group we could also base on the complaints as described in the questionnaire.

### Three measures to assess treatment outcomes

We used three measures to assess the treatment outcomes: the ‘open question outcomes’, the ‘score list’ and the ‘bell score’.

### Open question about outcomes in part B of the questionnaire

In response to the open question about outcomes, 93 out of 95 patients described the therapeutic outcomes of the SSRI in their own words. We developed a guideline for rating the answers to this Open question outcomes. Answers were rated in 5 categories: strong improvement (4), good improvement (3), reasonably good improvement (2), moderate improvement (1) and no improvement (0). Two independent researchers not involved in the study applied this guideline to the open-ended question, in addition to one of the authors. Based on these 3 applications, the inter-rater reliability of the guideline showed "good reliability" (ICC = 0.74; Cronbach’s alpha = 0.89). We refer to this first effect measurement as ‘Open question outcomes’.

### Score list

As the second effect measurement patients were asked in Part A of the *questionnaire* to indicate the severity of 8 common PCS symptoms (brainfog, chronic fatigue, sensory overload, headache, palpitations, dyspnoea, muscle weakness and muscle pain), in addition to any "other symptoms," on a scale from 0 (not troublesome) to 10 (extremely troublesome). This severity score had to be completed before starting the SSRI. In Part B of the questionnaire, completed at least 4 to 6 weeks after starting the treatment, the severity of these complaints was scored again. The difference between the 8 scores before and the scores 4 to 6 weeks after the start of the treatment, is used as the second outcome measurement.

Of the 95 patients, 77 completed the Score List. If a patient did not experience a complaint, s/he did not score it. Eighteen patients expressed difficulty in scoring the list, partly because of the great variability in the severity of the complaints. The 18 patients who did not fill out the Score List did not differ from the 77 patients who did fill out the Score List in terms of the Open Question of Outcomes, as tested with a Bayesian Mann–Whitney U Test (see "Statistical analysis") for comparison of groups (BF = 0.63). We refer to this second effect measurement as "Score List".

### Bell score

As a third effect measurement, the Bell’s Functionality Score was used^45^. This scale is developed to assess functional ability in adult ME/CFS patients. Eleven statements describe patient status such as level of symptoms at rest and with exercise, activity level, and ability to perform work, travel and self-care. The scale is scored in units of 10 from 0 to 100. An example of a statement, score 20: *‘Able to leave house once or twice a week. Moderate to severe symptoms. Able to concentrate for one hour or less per day’.* And score 50: *‘Able to do about 4–5 h of work or similar activity at home. Symptoms mostly moderate. Daily rests required’.* We presented it to the patients in February 2023, so it was partly completed retrospectively. Eighty patients responded. The Bell score was completed three times: to score their condition retrospectively before their Covid-19 infection, just before treatment with an SSRI during PCS, and 4 to 6 weeks after treatment with an SSRI. Completion was not readily possible for 15 patients because of too much variation in symptom severity. The 15 patients who did not complete the Bell score did not differ from the 80 patients who did complete the Bell score in outcome on the "Open question outcomes" (BF = 0.27).

In 14 patients with an autoimmune disease, pre-existing ME/CFS or Dengue virus in the past history, the severity of fatigue before initiation of Covid-19 infection/PCS was also checked. This was reflected in their initial Bell score, on which they scored significantly lower than the rest (on average 83.5 vs. 96.3, respectively; BF = 87.5).

### Statistical analysis

The data from the questionnaire, score lists, and Bell scores in Excel were loaded into MATLAB R2022b for further analyses and for creating tables and results figures. For statistical analysis we used JASP (version 0.17.1), a software package for Bayesian statistics^46^. Bayesian hypothesis testing directly evaluates the strength of evidence for one hypothesis (H1) over the alternative (null) hypothesis (H0), and this evidence is quantified by the Bayes Factor (BF)^47^. A BF of 10 for example, indicates that H1 is 10 times more likely than H0, given the observed data. In contrast, a BF of 0.1 indicates that H0 is 10 times more likely than H1. Thus, in contrast to traditional statistics, this method allows to directly quantify the evidence in favour of the null hypothesis^48^, e.g., of no group difference. A BF of 1 to 3 reflects anecdotal evidence, 3 to 10 moderate evidence, 10 to 30 strong evidence, 30 to 100 very strong evidence, and 100 or higher extremely strong evidence in support of H1^47^. In all our Bayesian analyses we used the default model parameters and prior distributions as set by JASP.

We analysed outcome per symptom with a Bayesian repeated-measures ANOVA, with the two factors SSRI treatment (before vs. after) and symptom (8). For the outcome as captured by the Bell scores, we used a Bayesian dependent sample t-test to test for a difference in Bell score between ‘during PCS’ and ‘after SSRI treatment’.

The relationship between the 10 outcome measures (i.e., the open question outcomes, the 8 symptom scores, and the Bell score) was analysed using Spearman’s rank correlation across patients. Additionally, we computed the combined symptom scores (by summing the 8 individual symptom scores) and correlated this combined measure with the outcomes on the Open question outcomes and the Bell score.

## Results

### Demographic and clinical characteristics

The mean age was 43 ± 11.5 (SD; min–max: 21–72) and women were over-represented (male–female ratio 1:5.8). 76% were living with a partner and 62% had one or more children. Over three-quarters had higher education (NL population: over one-third). The percentage of patients working in healthcare (31%) or education (18%) was significantly higher (BF = 591 en BF = 558, respectively) than in the general Dutch population: of the 10.0 million employed, 1.4 million (14%) work in healthcare, and 577,000 (5.7%) in education. Covid-19 infection, except for three patients, was without hospitalization. Before initiation of an SSRI, patients had PCS for an average of 15 ± 8.1 months (min–max: 3–36), and they were usually severely impaired. 30–40% felt numb (a dissociative symptom) or despondent because of their PCS but were not depressed (DSM-5). Two patients did develop clinical depression (first episode) and two developed an anxiety disorder (DSM-5 criteria).

76 patients (80%, n = 93) had comorbidity which is a risk factor for PCS^1^. See Table 2. 46 patients (49.5%) had asthma or an allergy: hay fever, mug wort (variant of hay fever), house dust, nickel, cats, other pets, grapefruit, shellfish, birds, insect bites, amoxicillin, clamoxyl, vibramycin, mold, latex, perfume, make up, gluten, sun, kiwi, plasters, nuts, peanuts, and sometimes multiple allergies simultaneously. Nine patients had an autoimmune disease: Sjogren’s, rheumatoid arthritis, Cushing’s, hypothyroidism, lichen planus, celiac disease, and high anti-nuclear antibodies (ANA); three had ME/CFS; two patients had had Epstein-Barr virus and one Dengue virus; ten had a connective tissue disorder: fibromyalgia, CRPS I, hemihypertrophy, and osteoarthritis. Seven had a psychiatric disorder, including three with AD(H)D, two with depression, and two with anxiety or panic disorder. Two patients had factor V Leiden thrombophilia.

**Table 2:** Demographic and clinical characteristics and response to SSRI.

	Total (n = 95)	Open question outcome (n = 93)					Group differences
		0 (n = 9)	1 (n = 25)	2 (n = 25)	3 (n = 27)	4 (n = 7)	
Age (min–max)	43.5 (21–72)	47.7 (25–72)	44.3 (21–68)	43.1 (22–62)	40.7 (25–58)	46.3 (30–57)	No (BF = 0.13)
Gender (f/m)	80/15	8/1	19/6	21/4	24/3	6/1	~ (BF = 0.34)
Hospitalization during COVID	3 (3.2%)	3 (33.3%)	0 (0%)	0 (0%)	0 (0%)	0 (0%)	Yes (BF = 16.4)
Medical history							
Auto-immune diseases	9 (9.5%)	1 (11.1%)	2 (8.0%)	4 (16.0%)	1 (3.7%)	0 (0.0%)	~ (BF = 0.48)
Asthma/allergies	46 (48.4%)	2 (22.2%)	14 (56.0%)	15 (60.0%)	12 (44.4%)	3 (42.9%)	No (BF = 0.22)
Pfeiffer/Dengue	3 (3.2%)	0 (0.0%)	0 (0.0%)	0 (0.0%)	3 (11.1%)	0 (0.0%)	~ (BF = 1.05)
Connective tissue disorders	10 (10.5%)	1 (11.1%)	5 (20.0%)	3 (12.0%)	1 (3.7%)	0 (0.0%)	~ (BF = 1.05)
ME/CVS	3 (3.2%)	0 (0.0%)	1 (4.0%)	1 (4.0%)	1 (3.7%)	0 (0.0%)	~ (BF = 0.47)
Factor V Leiden Thrombophilia	2 (2.1%)	0 (0.0%)	1 (4.0%)	1 (4.0%)	0 (0.0%)	0 (0.0%)	~ (BF = 0.58)
Psychiatric disorders: anxiety, panic, depression, or AD(H)D	7 (7.4%)	1 (11.1%)	0 (0.0%)	3 (12.0%)	2 (7.4%)	0 (0.0%)	~ (BF = 0.38)
Time gap in months between start PCS and SSRI (min–max)	15 (3–36)	20 (9–34)	16 (3–31)	14 (3–36)	14 (6–33)	15 (3–26)	No (BF = 0.18)
SSRI type: sigma1 agonists							
Citalopram	46 (48.4%)	4 (44.4%)	10 (40.0%)	17 (68.0%)	13 (48.1%)	2 (28.6%)	No (BF = 0.096)
Fluvoxamine	15 (15.8%)	2 (22.2%)	2 (8.0%)	3 (12.0%)	2 (7.4%)	4 (57.1%)	
Venlafaxine	17 (17.9%)	2 (22.2%)	7 (28.0%)	2 (8.0%)	5 (18.5%)	1 (14.3%)	
Escitalopram	10 (10.5%)	1 (11.1%)	4 (16.0%)	1 (4.0%)	4 (14.8%)	0 (0.0%)	
Fluoxetine	2 (2.1%)	0 (0.0%)	1 (4.0%)	0 (0.0%)	1 (3.7%)	0 (0.0%)	
SSRI type: Sigma 1 antagonists							
Sertraline/Paroxetine	5 (5.4%)	0 (0.0%)	1 (4.0%)	2 (8.0%)	2 (7.4%)	0 (0.0%)	

The column ‘Group differences’ indicates the amount of evidence for a group difference (BF > 1) or no group difference (BF < 1) between the 5 groups on the respective characteristic. ~ indicates no strong evidence in either direction (i.e., BF between 1/3 and 3). The effect of SSRI type on open question outcome is tested in a single Bayesian ANOVA. Note that one patient initially used sertraline (outcome 0) but switched to citalopram (outcome 2).

On the Score List (n = 77), 100% of patients reported brainfog and fatigue and 98.7% reported sensory overload. Headache and palpitations were reported in 90.9 and 88.3%, respectively. Muscle weakness occurred in 85.7%, muscle pain and spasm in 80.5%. On the open question about PCS symptoms, 100% reported PEM (n = 95). Fourteen patients reported severe dissociative symptoms such as derealization or depersonalization. One patient phrased it as follows: “I wake up every morning with no memory”.

### Reported outcomes

#### Open question about outcomes

**Figure 2:**
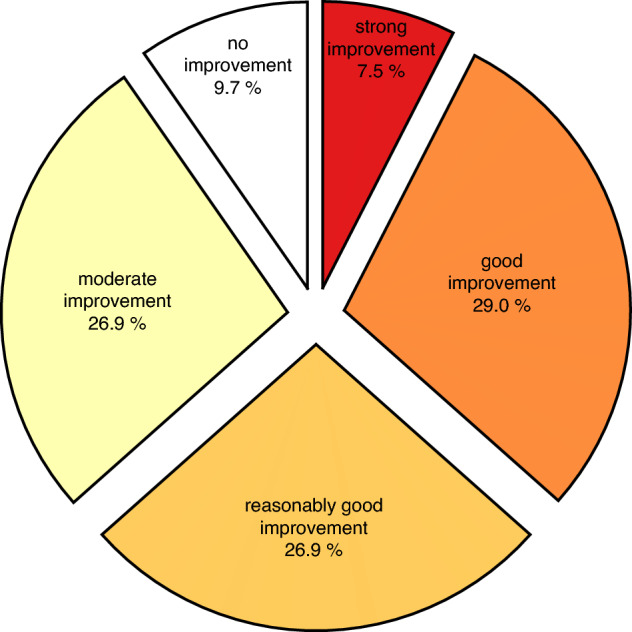
Open question outcomes: improvement as captured by the open question (n = 93).

63.4% of patients (n = 93) reported decrease in symptoms after treatment with an SSRI with an improvement that was reasonable good (26.9%), good (29%), and strong (7.5%) (see Fig. 2). 29 patients (31.1%) reported improved sleep. 67 patients (72.0%) described a decrease in PEM. Four patients reported decreased gastrointestinal symptoms. In one patient, fever had disappeared, and one patient was able to chew better again. In one patient, PCS had caused her only functioning adrenal gland to fail, for which she was treated with hydrocortisone. After an SSRI, her adrenal gland recovered and she was able to taper off the hydrocortisone dosage. Furthermore, in this patient, PCS increased her TSH from 2.5 mlU/l to 5.5 mlU/l (N 0.3–4.2 mlU/l). As a result, her free T4 increased from 14 pmol/l to 19 pmol/l (high-normal). After treatment with an SSRI, her TSH and free T4 dropped back to normal. The fourteen patients with dissociative symptoms reported that these had disappeared. Finally, it is noticeable that patients often report that the SSRI produces an increase in response in the months after starting. 24 patients who took the SSRI for more than six months reported that the outcomes were maintained.

There was no evidence in favour of or against a difference in outcome measured by the open question depending on whether patients received sigma1 agonists or antagonists (BF = 0.43). If we include the patient who changed from sertraline (no improvement) to citalopram (with reasonable good improvement), no significant difference remains (BF 0.39).

#### Score list: improvement per complaint

**Figure 3:**
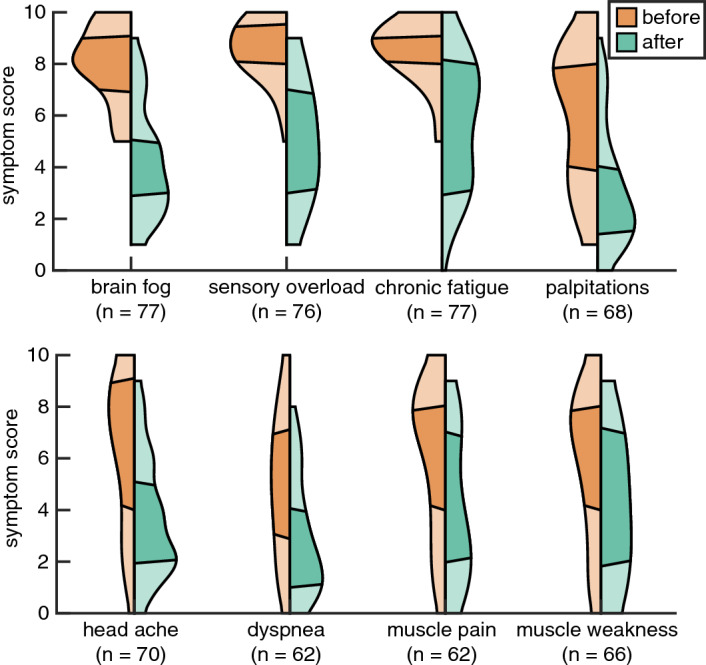
Improvement per complaint. Before (orange) and after (green) start of SSRI in violin plots per complaint. Dark segments indicate 50% of patients, while upper and lower light segments indicate the highest and lowest 25%, respectively. See x-axis label for symptom and number of patients (n = x) who scored that respective complaint.

The main finding is that there is strong statistical evidence for a positive effect on PCS when treated with an SSRI (Fig. 3). This finding is supported by a Bayesian variation of an ANOVA with the two factors SSRI treatment (before/after) and complaint (8), which indicates that there is strong evidence for a significant effect of SSRI treatment (BF = 5.0 × 1010 times greater for the model with SSRI treatment than the model without). Moreover, there is strong evidence for an interaction effect (BF = 64.6 times greater for the model with the interaction), implying that the effect of SSRI treatment varies by complaint. Brainfog and sensory overload decreased the most (3.8 and 3.6 pts, respectively), muscle pain and weakness responded the least (2.1 and 1.9 pts, respectively).

Patients with fibromyalgia (n = 5), in which muscle pain is the main symptom, reported little or no improvement after using an SSRI. Among other complaints, eight patients (10.4%, n = 77) reported a large decrease in PEM by an average of over 5.1 points. In 59 (76.6%) patients, this effect is indirectly reflected in the decrease in palpitations, fatigue and shortness of breath. Eight of 14 patients with tinnitus reported that this complaint diminished, in four this did not happen and for one the complaint worsened.

#### Bell score: improvement in functioning

**Figure 4:**
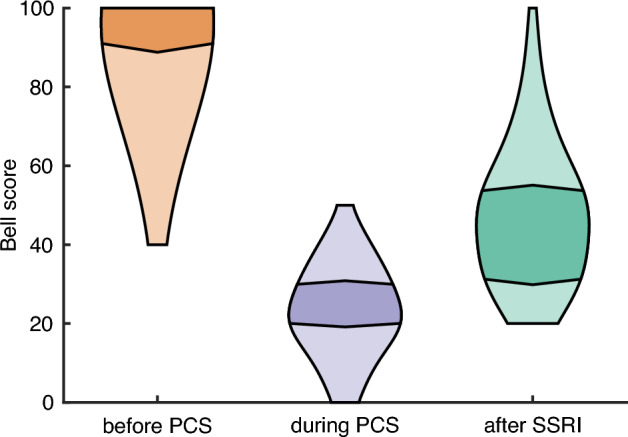
Bell scores. Bell scores before infection with Covid-19 and PCS, during PCS and after treatment with an SSRI in different violin plots (n = 80). The interval within which 50% of patients fall is represented by the darker areas, whereas interval within which the highest and the lowest 25% of patients fall is represented by the lighter areas.

As with the difference scores on the 8 complaints, we also find strong statistical evidence for the positive effect of an SSRI on the Bell scores (BF = 9.6 × 1017). There is a strong decline in functioning after infection with Covid-19 and PCS, from an average of 94.2 to 23.5 on the Bell Functionality Score: from healthy living and being able to work to severe disability, where a Bell score of 10 or lower implies being bedridden (see Fig. 4). This difference is supported by a Bayesian variant of a t-test (BF = 9.1 × 1052). Treatment with an SSRI gives an average increase to 47.2: to full self-care, shopping, walking, and (partial) return to work. While this may seem like little progress on the full scale, it is important to note that the Bell score is not linear: the greatest subjective improvements occur up to score 50.

Of the 36 patients who had used an SSRI for 2 months or less, 17 (47.2%) reported that they still noticed reduction in symptoms after the measurement. In one patient this was still true after 4 months, in one patient after 6 months and in one patient after 7 months.

### Relationship between the three effect measures

**Figure 5:**
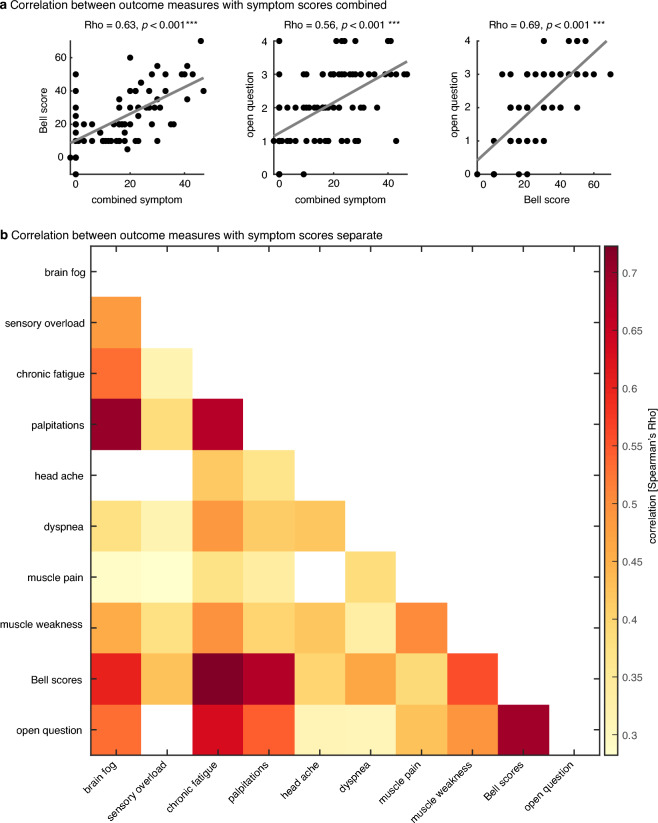
Relationship of effect measures. (**a**) Relationship between the three effect measures (Spearman’s Rho), combining the 8 complaint scores. Dots represent individual patients. (**b**) The same correlation results but with the 8 complaints separately. Dark colours represent high correlations. Only significant correlations are shown (i.e., correlations with a *p* value > 0.05 are coloured white).

The three different effect measures show a highly significant correlation among themselves (see Table 3 and Fig. 5 for pairwise Rho and *p* values). That is, patients who improve strongly on one effect measure (e.g. brainfog) also improve strongly on most of the other effect measures (e.g. sensory overload), and vice versa. It is interesting to note that both Bell scores and the Open question outcomes correlate strongly with the various complaint-specific improvements (except headache with the Open question outcomes). This is further supported by a highly significant correlation between the combined improvement on all eight complaints and the Bell scores (Rho = 0.63, *p* = 5.5 × 10^−10^; left panel in Fig. 5a), and a highly significant correlation between this combined improvement and the Open question outcomes (Rho = 0.56, *p* = 6.4 × 10^−9^ middle panel in Fig. 5a). Additionally, the Open question outcomes also correlated strongly with the Bell score (Rho = 0.69, *p* = 1.4 × 10^−12^; right panel in Fig. 5a). The fact that the three outcome measures correlated strongly with each other supports their individual reliability.

**Table 3:** Relationship effect measures.

	Brainfog	Headache	Chronic fatigue	Over-stimulation	Dyspnea	Palpitations	Muscle pain	Muscle weakness	Bell score	Open questio
Brainfog										
Headache	Rho = 0.48 *p* < 0.001***									
Chronic fatigue	Rho = 0.53 *p* < 0.001***	Rho = 0.32 *p* = 0.008**								
Over-stimulation	Rho = 0.70 *p* < 0.001***	Rho = 0.39 *p* < 0.001***	Rho = 0.67 *p* < 0.001***							
Dyspnoea	Rho = 0.25 *p* = 0.052	Rho = 0.15 *p* = 0.261	Rho = 0.42 *p* < 0.001***	Rho = 0.37 *p* = 0.003**						
Palpitations	Rho = 0.38 *p* = 0.002**	Rho = 0.32 *p* = 0.011*	Rho = 0.49 *p* < 0.001***	Rho = 0.41 *p* < 0.001***	Rho = 0.42 *p* < 0.001***					
Muscle pain	Rho = 0.29 *p* = 0.023*	Rho = 0.28 *p* = 0.028*	Rho = 0.37 *p* = 0.003**	Rho = 0.34 *p* = 0.007**	Rho = 0.13 *p* = 0.357	Rho = 0.39 *p* = 0.003**				
Muscle weakness	Rho = 0.46 *p* < 0.001***	Rho = 0.38 *p* = 0.003**	Rho = 0.49 *p* < 0.001***	Rho = 0.40 *p* < 0.001***	Rho = 0.42 *p* = 0.001**	Rho = 0.33 *p* = 0.010**	Rho = 0.51 *p* < 0.001***			
Bell scores	Rho = 0.60 *p* < 0.001***	Rho = 0.42 *p* < 0.001***	Rho = 0.72 *p* < 0.001***	Rho = 0.68 *p* < 0.001***	Rho = 0.40 *p* = 0.003**	Rho = 0.47 *p* < 0.001***	Rho = 0.39 *p* = 0.003**	Rho = 0.56 *p* < 0.001***		
Open question	Rho = 0.53 *p* < 0.001***	Rho = 0.16 *p* = 0.194	Rho = 0.63 *p* < 0.001***	Rho = 0.55 *p* < 0.001***	Rho = 0.31 *p* = 0.016*	Rho = 0.30 *p* = 0.013*	Rho = 0.43 *p* < 0.001***	Rho = 0.49 *p* < 0.001***	Rho = 0.69 *p* < 0.001***	

Relationship between the three effect measures (Spearman’s Rho), where the number of stars reflects the significance level: *p < 0.05, **p < 0.01, ***p < 0.001.

### Side effects

When taking an SSRI for depression or anxiety, patients generally have side effects for the first few weeks, after which the response begins. Patients in this study were educated about this. 30 patients (31.6%, n = 95) initially experienced serious side effects, 52 (54.7%) experienced mild, and 13 (13.7%) no side effects. The side effects malaise, headache and dizziness were similar to PCS symptoms. 72 patients (75.7%) experienced no effect but side effects in the first few weeks. In 65/82 (79.2%) patients with side effects, these reduced or disappeared within a few weeks.

### Non-responders

The 9 patients (9.5%, n = 95) with no response showed no difference in age, risk factors or PCS symptom pattern compared with the other groups. There was a difference in severity of infection with Covid-19: the group that did not improve with an SSRI seemed to have had worse initial symptoms than the other groups based on the open question about symptoms during the Covid-19 infection. Three of them were hospitalized because of severe pneumonia, double pulmonary embolism and thrombosis. Also, the 6 patients from the non-responders group who were not admitted described a more severe illness due to Covid-19 than the groups that did respond to an SSRI.

## Discussion

The results of this exploratory questionnaire-based study of the effect of treatment with an SSRI in PCS show that an SSRI contributes significantly to the reduction of PCS symptoms. 63.4% (n = 95) of patients reported a reasonably good to strong decrease in symptoms and an improvement in functioning. This increased quality of life can contribute to social participation. Four patients had developed clinical depression or anxiety disorder during PCS. Treatment with an SSRI also eliminated these disorders. People who had felt despondent due to PCS also felt less gloomy after an SSRI, but all attributed this to the reduction in their PCS symptoms.

An explanation for the skewed gender distribution in this study—also found in other research in PCS—may be that many genes for the immune system lie on the X chromosome so that men are more likely to have severe Covid-19 infections, but women are more likely to have more severe PCS symptoms that last longer^7,49^.

The high level of education in this study compared to other studies^1^ can probably be explained by the recruitment of patients through LinkedIn. The overrepresentation of female patients from healthcare and education could be caused by the predominance of female workers in these professions on the front lines of the pandemic. Moreover, they returned to work quickly after their Covid-19 infection which is a risk factor for PCS^1^. Many other risk factors for PCS in this study are consistent with Davis’s review article^1^, such as: asthma, allergies, connective tissue diseases, Epstein-Barr virus etc. A notable addition to the known risk factors is factor V Leiden thrombophilia. In this study, there were two patients with this coagulation disorder, while the prevalence in the general population is only 1:5000^50^. Patients with this coagulation disorder have an 80-fold increased risk of thrombosis.

All patients in our group (n = 95) were chronically fatigued during PCS while in a German study^11^ only 19/42 patients of the PCS study population reported chronic fatigue such as in ME/CFS. An explanation for this difference could be that fatigue in PCS increases with time. While the duration of PCS in the German study was only six months, in our study it averaged 15 months. Also, in this German study, of the other PCS patients without chronic fatigue (23/42), only 15 had a neurological or cognitive impairment. In our study, 100% had a neurological or cognitive impairment. However, neurocognitive symptoms begin only a month to a few months after Covid-19 infection and worsen over time^51^.

### Strength and weakness of the study

No validated questionnaires are yet available for PCS. However, with a new disease, it is important to learn about all symptoms, so we used a questionnaire that included open-ended questions. Thus, we discovered that symptoms can shift over time. Dyspnoea and decrease in smell seem to decrease over time, while fatigue and brainfog seem to increase. Through the open-ended questions we also discovered new symptoms, such as dissociatieve symptoms and not being able to chew properly.

Using three different instruments to determine treatment effectiveness is a further strength of this study. There is strong evidence for the reliability of these measures. The Bell score is a widely used instrument in research on (chronic) fatigue, although not validated. Furthermore, the rating of the Open question outcomes was found to be reliable. Importantly, the three effect measures correlated strongly with each other, supporting the reliability of the individual measures.

The main weakness of this study is that it is not a randomized controlled trial (RCT). We had no control group. Therefore, a placebo effect cannot be ruled out. However, it is known that 85% of patients who have symptoms two months after Covid-19 infection still have them after one year^1^. ME/CFS and dysautonomia are usually lifelong^1^. So without treatment with an SSRI, many PCS patients may suffer from these conditions in permanently.

And there is more evidence that a placebo effect may not fully explain the positive results. A placebo effect usually occurs shortly after the start of an intervention and diminishes again after a few weeks, unless a positive expectation is given again^52^. However, 72 patients (n = 95) still had no response in the first weeks, but instead suffered side effects. The 24 patients who used the SSRI for more than six months reported that the effect was maintained, while they were not asked by us to do so, as we had no treatment relationship with the patients. A cohort study without a control group also does not exclude natural recovery. However, because the participants had been seriously ill for 1.5 years and deteriorated over time, it seems highly unlikely that, if they had received an SSRI and recovered after a few weeks, this would have been due to natural recovery.

Working with self-reporting is always vulnerable to biases. However, self-report often expresses the experience of patients better. In psychiatry not all physiological parameters can be measured. We also asked the patients to rate their complaints on a scale of 1 to 10. By inviting them to compare the severity of their complaints, we introduced more structure into the self-report.

Finally, low-cost SSRIs are covered by insurance and there is no cost to the patient, which argues against a placebo effect^53^. However, the possibility of a placebo effect can only be completely ruled out by an RCT.

The Bell score before Covid-19/PCS and the Bell score during PCS before starting an SSRI were completed retrospectively by all patients. This may have led to some bias. The complaint of PEM was not part of the 8 complaints in the Score list; patients had to list and score this on their own under "other complaints". This may have led to underreporting of this important symptom. LinkedIn gave an overrepresentation of patients with a higher level of education, but perhaps also of a group of initially healthy people who were fully employed. It is precisely in this group that the impact of PCS as well as the outcomes of an SSRI may be well observed.

### Potential mechanisms of action of SSRIs in PCS

#### Dysregulation of the tryptophan system

**Figure 6:**
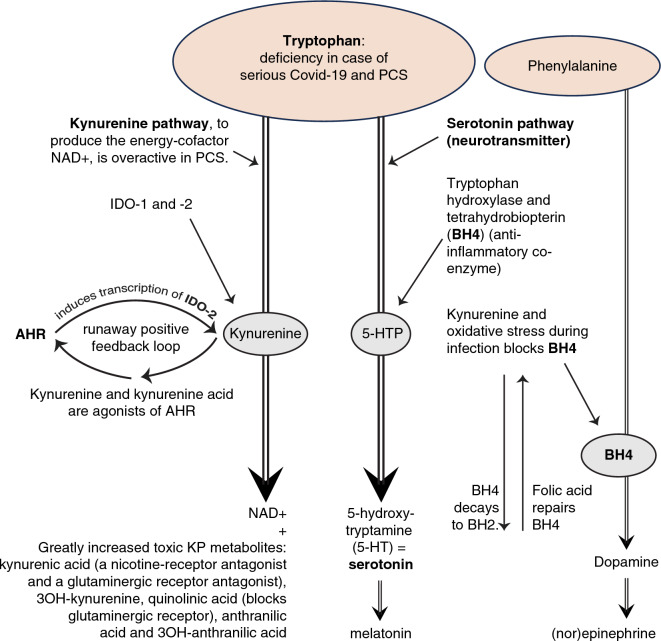
The overactive KP affects the serotonin pathway. The aryl hydrocarbon receptor (AHR) has a role in regulating immunity and induce transcription of indoleamine 2,3-dioxygenase (IDO-2) enzyme. The amount of IDO-2 in PCS is abundant and causes autophagy and reduced mitochondrial functioning^28^. Because the metabolites kynurenine and kynurenine acid are agonists of the AHR receptor, there is a ‘runaway positive feedback loop’ producing more and more metabolites^28,30–32^. Several of the metabolites are potentially toxic. Kynurenine and oxidative stress block the enzyme tetrahydrobiopterin (BH4)^1,56^, inhibiting both the serotonin pathway and the dopamine pathway^1,20,55,56^. Kynurenic acid is a nicotine-receptor antagonist and a glutaminergic-receptor antagonist^28,30,32^. Quinolinic acid blocks the glutaminergic receptor^28^. When we want to intervene: the kynurenine pathway can be inhibited in various ways and the serotonin pathway can be stimulated in various ways. Intervening through an SSRI has been the most important so far.

Normally the catabolic kynurenine pathway (KP) degrades 95% of the essential amino acid tryptophan to produce the vital energy cofactor NAD+. The rest of the tryptophan serves as a precursor for serotonin and melatonin. (See Fig. 6). In addition to NAD+, the KP generates different metabolites: kynurenine, kynurenic acid, 3OH-kynurenine, quinolinic acid, anthranilic acid and 3OH-anthranilic acid. The KP is stimulated by inflammation and in PCS it is found overactive^26–28,30,32^.

The overactive KP absorbs more than 95% of the tryptophan in PCS. Research shows that the amount of tryptophan in blood is decreased in PCS patients^30–32^. Tryptophan deficiency is probably already present during the infection with Covid-19, because Covid-19 attaches with its spike proteins to the Angiotensin Converting Enzyme (ACE2) receptors in the intestines, while tryptophan also uses the same receptor to be absorbed from the intestines^54^. There is a significant relationship between the level of metabolites in blood and the severity of cognitive impairment in PCS^28^, (*p* < 0.001)^30,31^. The KP has normally potentially neurotoxic as well as neuroprotective aspects. An overactive KP during a severe infection however, is toxic for neurons, especially for serotonin neurons. Not only does the KP hijack tryptophan away from the serotonin pathway^28,30,31^, the metabolite kynurenine and oxidative stress also lowers the level of tetrahydrobiopterin (BH4)^1,55,56^, an important coenzyme of the serotonin pathway. Moreover, BH4 is an important coenzyme of the dopamine pathway—and to the norepinephrine pathway^20,55^—thus the overactive KP damages these two neurotransmitter systems too. When there is no longer enough tryptophan, this can lead to serotonin depletion^16^.

SSRI’s make the serotonin in the neurons more available through inhibition of reuptake and can partly compensate for the deficiency of tryptophan^57^. Moreover, SSRIs lower oxidative stress^58^. That could be an explanation for our finding that PCS patients often have fewer complaints due to SSRIs. But that may come to an end when all the serotonin from the neurons is in the synapses. Nevertheless, after half a year or more (in this research 24 patients), many people still felt good when using an SSRI. So we can (hypothetically) conclude that an SSRI probably indirectly contributes to—when there is a lack of serotonin in the neurons—that these neurons start to pull harder on the available tryptophan. So SSRIs could slow down the overactive KP. There is no theoretical argument against this hypothesis^16^.

But there are more avenues for future research advancing this line of clinical research based on tryptophan metabolism. IDO2 expression can be halted by an AHR antagonist^28^, such as the dietary supplement resveratrol^59^ or the experimental anticancer drug IK-175^60^. Furthermore, kynurenine is a glutaminergic receptor antagonists and quinolinic acid even blocks this receptor^28^. N-acetylcysteine (NAC) not only produces the antioxidant glutathione, but is also a glutaminergic receptor agonist^61^. The poisonous quinolinic acid and kynurenine acid are nicotinic receptor antagonists. Nicotine is a nicotinic receptor agonist. To stick nicotine patches helps PCS patients. This may be not only because nicotine is a nicotinic receptor agonist and therefore an opponent of these poisonous metabolites, but nicotine is a strong acetylcholine (ACh) agonist as well^62^. ACh is the most important neurotransmitter in muscles. Furthermore, folic acid (vit. B11)^63^ can promote the conversion of B2 back into B4^55,63^ in favour of the serotonin and dopamine pathway.

#### Disrupted HPA axis

After a stressful event, the hypothalamus secretes corticotropin-releasing hormone (CRH), which causes the pituitary gland to secrete adrenocorticotropic hormone (ACTH), which in turn causes glucocorticoids (GCs) to be released by the adrenal cortex.

In PCS cortisol (glucocorticoids) levels were on average halved^19^. That is an indication that the HPA axis is disrupted^14,19^. In comparison, in ME/CFS, the HPA axis is less severely disrupted^12^. GCs released by the adrenal glands, act on almost all types of immune cells and perform evident immunosuppressive and anti-inflammatory functions^14–18^. SSRIs affect the HPA axis^16,64^. For example by increasing GC receptor density in the hypothalamus and hippocampus (the memory control centre). Further on, acute administration of 5-HTP receptor ligands increased the plasma levels of ACTH and cortisol in both animals and humans^15,16^. But the effect is not one-dimensional. For example: in patients with major depression with high cortisol levels, an SSRI lowers the cortisol level but triggers a higher Cortisol Awaking Response (CAR)^14–17,19^. In ME/CFS, an SSRI works moderately in only one-third of patients^12^. SSRIs seem to work better in PCS than in ME/CFS. This may be an indication that SSRIs are (partly) effective in PCS by influencing this hormone axis. Another indication of this hypothesis is our report of the patient who was given hydrocortisone because PCS caused her only functioning adrenal gland to fail, but who recovered with an SSRI, after which she was able to taper off the hydrocortisone. In this patient, the SSRI apparently restored not only the HPA axis, but also the disrupted hypothalamic-pituitary-thyroid axis^15,16^. The question is therefore whether—apart from the HPA axis—more hormonal axes starting from the hypothalamus are disturbed by Covid-19/PCS. Thus, reported changes in the menstrual cycle after Covid-19^65^ could also result from a disrupted hypothalamic-pituitary–gonadal axis.

#### Disrupted brain stem

The brain stem, the oldest part of our brain, is responsible for basic functions such as body temperature, sleep–wake rhythm, heart rate, breathing, blood pressure, digestion, eye movements, urination, hearing, tasting, chewing, swallowing, and feeling movement and gravity. Neurotransmitters that are especially important there include serotonin, norepinephrine and dopamine^10^. The serotonergic neurons start in the raphe nuclei in the pons and may exert their influence there^10^.

The Covid-19 virus enters the brain stem cells easily attaching to the many ACE2 receptors^66^. Hypometabolic areas in the pons were found in a study of three PCS patients. Typically, SSRIs worsen sleep quality^67^, though, not always^68^. In this study, 29 patients reported in the open-ended question that their sleep improved on the SSRI. This could be explained by the influence of the SSRI on the brainstem. But this may not be the only reason that patients sleep better. We saw in Fig. 6 that the sleep hormone melatonin originates from serotonin. When the serotonin neurons withdraw more from the available tryptophan, more melatonin can be produced in addition to more serotonin. The decrease in palpitations, shortness of breath, gastrointestinal complaints, better temperature regulation (one patient) and the ability to chew better (one patient) also supports the idea that SSRIs may (partially) restore the neurotransmitter systems disrupted in the brainstem.

#### Disrupted autonomic nervous system (ANS) balance

Dysautonomia, especially POTS, is often a symptom of PCS. The clinical picture with a Bell score of 20 or less (almost half of the patients in this study) is striking, as shown by the answers to the open questions: overstimulated patients with palpitations lying limp and exhausted on the couch. In this state, the brainstem shows heightened arousal and seems to be in the fight-or-flight response along with the sympathetic nervous system^14,69^. This is a primitive survival mechanism in the face of danger, in which the sympathetic ANS dominates. The 'fight-or-flight' response activates the HPA axis so that extra cortisol is released and glucose is released into the muscles for action^69^. Instead, measured cortisol levels in PCS patients average only 50% of normal^19^ and the muscles are weak rather than ready to contract. The muscle weakness is not only because no glucose is released in the muscles. It is also because PCS is associated with autoantibodies against the G protein-coupled adrenergic receptor and muscarinic acetylcholine receptor^70^. Acetylcholine is the main neurotransmitter that sets muscles in motion.

The vagus nerve of the (parasympathetic) ANS also originates from the pons in the brainstem. But this nerve—just like muscles—uses acetylcholine as a neurotransmitter, so an SSRI cannot intervene there. One speaks of a paralyzed vagus nerve, probably because of the autoantibodies against the muscarinic acetylcholine receptor^1^. The sympathetic ANS predominates over the parasympathetic ANS in PCS, while G protein-coupled autoantibodies against the adrenergic receptor are also present. Perhaps the (nor)epinephrine pathway has fewer problems with the overactive KP, because it does not use tryptophan as a precursor, but phenylalanine. SSRIs often seem to reduce POTS and palpitations. This is unrelated to an inhibition of serotonin reuptake, as serotonin does not affect the ANS. The inhibition of norepinephrine reuptake may reduce POTS, but could theoretically lead to palpitations. The reduction in palpitations must be found in another mechanism, such as the influence of SSRIs on the anterior cingulate cortex (ACC) in the brain^61^.

#### CNS symptoms

Brainfog and sensory overload responded best to treatment with an SSRI. The raphe nuclei (pons) in the brain stem is the location of the origin of the serotoninergic system. From there, axons are sent throughout the CNS^10^. So SSRIs can intervene throughout the whole brain.

Dissociative symptoms also disappeared. In sensory overload and dissociation, there is sensory overload due to lack of filtering. The primary unimodal sensory brain regions do not cooperate well with the associative sensory brain regions^71,72^. It is known that an SSRI can sometimes help with this^71,73^.

Many PCS patients struggle with forgetfulness^1^. In the hippocampus, the control centre of memory, serotonergic neurons are dominant^74^ SSRIs also stimulate the production of serotonin cells in the hippocampus^74^. Possibly partly because of this, the patients’ forgetfulness decreased.

#### Sigma1 receptor agonist

The SSRIs fluvoxamine and fluoxetine have been shown to have extra anti-inflammatory effects during Covid-19 infection by inhibiting sphingomyelinase acid (ASM)^33^. Furthermore, an SSRI reduces the pro-inflammatory cytokines Interleukin 2 (IL 2) and IL 17 in the CNS. In this case, the SSRI must be a sigma1 receptor agonist. This opioid receptor is inter alia involved in reducing virus replication and inhibiting reactivation of herpes viruses such as Epstein-Barr Virus (EBV) and subsequent endoplasmic reticulum (ER) stress and inflammation^35^. We recommended only SSRIs who are sigma1 receptor agonists^34,35^. One patient was first given sertraline by the GP, with no response. After switching to citalopram, she did respond reasonable good. In five other patients, GPs prescribed the sigma1 receptor antagonists sertraline (n = 4) or paroxetine (n = 1). These five patients reported good (n = 2), reasonable good (n = 2) or moderate (n = 1) improvement. There was no evidence in favour of or against a difference in effect measured by the open question depending on whether patients received sigma1 agonists or antagonists (BF = 0.43). If we include the patient who changed from sertraline (no effect) to citalopram (reasonable good effect), no significant difference remains (BF = 0.39) This is only anecdotal evidence that the mechanism via the sigma1 receptor plays a role in the action of SSRI in PCS. However, the group using a sigma1 antagonist in this study is too small for a proper statistical analysis.

#### Positive influence of SSRIs on the circulatory system

Many Covid-19 and PCS patients have microclots indicative of coagulation problems. Microclots impede oxygen and nutrients flow to organs and tissues^8^. Platelets are involved in clotting. Platelets transport serotonin, because serotonine has a function in clotting. With serotonin deficiency, platelets become less functional. Because SSRIs inhibit the reuptake of serotonin in platelets, they prolong clotting time and could theoretically dissolve microclots^75,76^. The two patients with factor V Leiden thrombophilia responded well and moderately, respectively. This could mean that the anticoagulant effect of SSRIs—assuming that microclots played a role in these patients—might contribute to their response. If it were confirmed that PCS is more common in factor V Leiden thrombophilia, this coagulation disorder should be added to the list of risk factors.

But an SSRI can benefit blood circulation in other ways, too. They may show an anti-inflammatory effect on endothelial cells. SSRIs reduce the expression of cytokine-induced endothelial adhesion. This makes it difficult for circulating adhesion molecules, such as monocytes, to adhere^77^. This mechanism may partially explain their cardioprotective effects^76^.

### Subgroup with fibromyalgia

Muscle pain and weakness decreased the least in the total group (n = 95). We also see this in the patients with fibromyalgia (n = 5) who reported little or no improvement after using an SSRI.

### Non-responders

The group of non-responders (n = 9) were more severely ill with Covid-19 infection than those who did report good response to SSRIs. It is possible that the cascade of severe inflammation caused by Covid-19 in the CNS^78^ released much histamine. Inhibitory histamine receptors lower serotonin in the CNS, preventing an SSRI from effectively releasing extracellular serotonin^40^. By adding Histamine1 and 2 antagonists to treatment, an SSRI could in principle still become effective^40^. However, the lack of response to an SSRI may also have been caused by a serious factor: namely, neuropathology similar to Alzheimer’s disease is found in Covid-19 infections. In the CNS β-amyloid aggregations, plaque formations, tauopathy and cell death have been described^79^. In these conditions, an SSRI cannot possibly be effective anymore.

### When should the SSRI be phased out?

Our hypothesis is that for many PCS symptoms, neurotransmitter systems are not damaged but dysregulated and most likely suffering from a tryptophan deficiency due to an overactive KP. But after a chronic course of PCS lasting two years, SSRIs cannot be expected to ”reset” these systems in a short time as long as the kynurenine pathway is still overactive. By comparison, the treatment duration for initial depression is six months to one year and for recurrent depression usually at least 3 years^80^. In toxic drug-induced depersonalization disorder, sometimes 6 years of treatment is advised (first author’s clinical experience)^73^. Drug-induced depersonalization disorder is phenomenologically similar to PCS, excluding fatigue and muscle pain. Poor stimulus selection, sensory overload, derealization and brainfog are similar in the two conditions. The preliminary recommendation is to continue treatment with an SSRI for at least 1.5—2 years. More research is needed to support our hypothesis regarding the resetting of neurotransmitter systems by SSRIs. Another possibility could be that SSRIs only suppress symptoms because of the tryptophan deficiency. Contrary to this is the experience of two patients (the first one with a very low Bell score of 20 at the start of SSRI treatment) who discontinued the SSRI after 8 months and a year, respectively, because they felt completely healthy for 2 and 7 months, respectively. They continue to do well (seven and four months after discontinuation). However, most patients mention that, despite the good response to the SSRI, they must be cautious of not exceeding their limits. The PEM had become much less, but with too many activities in a row, a relapse—albeit shorter and less severe than before—could follow.

## Conclusion

This first exploratory questionnaire study of SSRIs in PCS patients shows that this treatment can contribute to considerable reduction of symptoms, especially brainfog and sensory overload. Fatigue, POTS, palpitations, PEM and overall functioning also improved significantly. In the absence of any other completed research on effective medicinal treatment of PCS, this is a meaningful result**.** If further confirmed, this could be of important personal and societal impact.

We formulated seven possible explanatory neurobiological mechanisms based on recent literature on PCS integrated with already existing insights. Clinical evidence for five of the seven possible explanatory mechanisms of an SSRI in PCS were found in this study. Those are the influence on the HPA axis, brain stem, ANS, CNS and the influence on the circulation system. The dysregulation of the tryptophan system is probably the main underlying biochemical construct that largely explains these five mechanisms.

Perhaps the mechanism that sigma1 receptor agonists can prevent virus replication might also work, but the group who used a sigma1 receptor antagonist was too small to compare it with the big group who used an sigma1 agonist and we got only anecdotal evidence for it.

Probably it is a combination of a number of mechanisms by which an SSRI is effective in PCS. The mechanisms are only partially the same as those thought to be responsible for the improvement of depression or anxiety disorders.

An RCT with SSRIs in PCS patients remains an urgent follow up to this study. In that RCT the individual upward titration of the SSRI dosage should be ascertained. We do realize that this titration, based on individual responses and side effects, is difficult to combine with an RCT. Practical solutions should be found like creating a pharmacogenetic profile in advance, because many people break down certain SSRIs poorly. That study could also determine biomarkers such as cortisol, coagulation, KP metabolites and the cytokines elevated in PCS, both before and during SSRI use. Measurement moments 4 to 6 weeks after the start and after 3 to 6 months during the use of the SSRI are recommended.

In PCS different patient groups are distinguished^1^. These groups overlap partly: chronic fatigue, PEM, cognitive complaints, MCAS, POTS, muscle problems etc. With additional research, it is important to gain more clarity for which patient groups SSRI medication works best.

Furthermore, it would be useful to compare one group receiving an SSRI which is a sigma1 receptor agonist and another group receiving an SSRI which is a sigma1 receptor antagonist. Also the influence of some of the supplements and medications as NAC^61^, resveratrol^59^, IK-175^60^, folic acid^63^, nicotine plasters^62^—whether or not added to an SSRI—can be investigated.

Given the serious individual, social and economic consequences of PCS, further study is of great importance.

## Data Availability

The data files and the instruments used are available from the first author.
